# Aktuelle Praxis der empirischen Antibiotikatherapie bei Spondylodiszitis

**DOI:** 10.1007/s00132-022-04240-x

**Published:** 2022-04-07

**Authors:** Siegmund Lang, Nike Walter, Carsten Neumann, Susanne Bärtl, Michaela Simon, Martin Ehrenschwender, Florian Hitzenbichler, Volker Alt, Markus Rupp

**Affiliations:** 1 Klinik und Poliklinik für Unfallchirurgie, Universitätsklinikum Regensburg, Franz-Josef-Strauß-Allee 11, 93053 Regensburg, Deutschland; 2 Institut für Mikrobiologie und Hygiene, Universitätsklinikum Regensburg, Franz-Josef-Strauß-Allee 11, 93053 Regensburg, Deutschland; 3 Institut für Labormedizin, Mikrobiologie und Krankenhaushygiene, Krankenhaus Barmherzige Brüder Regensburg, Prüfeninger Str. 86, 93049 Regensburg, Deutschland; 4 Abteilung für Krankenhaushygiene und Infektiologie, Universitätsklinikum Regensburg, Franz-Josef-Strauß-Allee 11, 93053 Regensburg, Deutschland

**Keywords:** Deutschland, Koagulase-negative Staphylokokken, Antibiotic Stewardship, Therapiestandards, Bundesweite Umfrage, Germany, Coagulase-negative staphylococci, Antibiotic Stewardship, Therapy Standards, Nationwide Survey

## Abstract

**Hintergrund und Fragestellung:**

Bei der pyogenen Spondylodiszitis gewinnen Infektionen mit Koagulase-negativen Staphylokokken zunehmend an Bedeutung. Eine empirische Antibiose ist insbesondere bei Patienten mit schweren oder progredienten neurologischen Ausfällen sowie hämodynamischer Instabilität und im Falle von kulturnegativen Spondylodiszitiden notwendig. Ob es in Deutschland einheitliche, an das Resistenzprofil angepasste Standards der empirische Antibotikatherapie gibt, ist unklar.

**Studiendesign und Untersuchungsmethoden:**

Es wurde an deutschen Universitäts- und berufsgenossenschaftlichen Kliniken, jeweils in den Fachbereichen Orthopädie und Unfallchirurgie, eine Umfrage zur empirischen Antibiotikatherapie bei pyogener Spondylodiszitis durchgeführt. Die Umfrageergebnisse wurden auf das Resistenzprofil der Erreger von 45 Spondylodiszitispatienten, die zwischen 2013 und 2020 in unserer Klinik behandelt wurden, angewandt. Dadurch wurden potenzielle Sensibilitäts- und Resistenzraten für die angegebenen antibiotischen Therapien errechnet.

**Ergebnisse:**

Von den 71 angefragten Kliniken antworteten insgesamt 44 (62,0 %). Sechzehn verschiedene Antibiotikatherapien wurden als jeweiliger Standard berichtet. Darunter wurden 14 verschiedene Kombinationstherapien als Therapiestandard angegeben. Die am häufigsten angegebenen empirischen Substanzen, nämlich Amoxicillin-Clavulansäure oder Ampicillin/Sulbactam (29,5 %) und Cephalosporine (18,2 %) zeigten in Bezug auf das zuvor veröffentliche Resistenzprofil hohe potenzielle Resistenzraten von 20,0 % bzw. 35,6 %. Die höchsten potenziellen Sensibilitätsraten wurden durch die Kombinationen Vancomycin + Ampicillin/Sulbactam (91,1 % sensible Erreger), Vancomycin + Piperacillin/Tazobactam (91,1 % sensible Erreger) und Ampicillin/Sulbactam + Teicoplanin (95,6 % sensible Erreger) erreicht. Eine dieser Kombinationen wurde von drei Kliniken (6,8 %) als Standard angegeben.

**Schlussfolgerung:**

Die deutschlandweite Umfrage zur empirischen Antibiose bei pyogener Spondylodiszitis hat eine große Heterogenität der Standardtherapien ergeben. Eine Kombination aus einem Breitspektrum-β-Laktam-Antibiotikum mit einem zusätzlichen Glykopeptidantibiotikum kann sinnvoll sein.

## Abstract (en)

**Background and research question:**

In pyogenic spondylodiscitis, infections with coagulase-negative staphylococci must be given increasing importance. Empirical antibiosis is particularly necessary in patients with severe or progressive neurological deficits or hemodynamic instability, as well as in the case of culture-negative spondylodiscitis. It is unclear whether uniform empirical antibiosis standards adapted to the resistance profiles exist in Germany.

**Study design and methods:**

A survey on the empirical antibiotic therapy for pyogenic spondylodiscitis was conducted at German university and Berufsgenossenschaft clinics, each in the departments of orthopedics and trauma surgery. The survey results were applied to the resistance profiles of pathogens in 45 spondylodiscitis patients treated in our department between 2013 and 2020. Thus, the potential susceptibility and resistance rates were calculated for the indicated antibiotic therapies.

**Results:**

Of the 71 clinics queried, a total of 44 (62.0%) responded. Sixteen different antibiotic therapies were reported as standard regimes. Among these, 14 different combination therapies were reported. The most commonly reported empirical antibiotics, namely amoxicillin/clavulanic acid or ampicillin/sulbactam (29.5%) and cephalosporins (18.2%) showed high potential resistance rates of 20.0% and 35.6%, respectively, in relation to the previously published resistance profile. The highest potential susceptibility rates were achieved with a combination of vancomycin + ampicillin/sulbactam (91.1% sensitive pathogens), vancomycin + piperacillin/tazobactam (91.1% sensitive pathogens), and ampicillin/sulbactam + teicoplanin (95.6% sensitive pathogens). One out of these combinations was reported as standard regime by three clinics (6.8%).

**Conclusion:**

The nationwide survey of empiric antibiotic treatment for pyogenic spondylodiscitis revealed a large heterogeneity in the standard of care. A combination of a broad-spectrum-β-lactam antibiotic with an additional glycopeptide antibiotic may be justified.

## Hintergrund und Fragestellung

Infektionen des Bewegungsapparates stellen ein große Herausforderung in der Orthopädie und Unfallchirurgie dar [1]. Nicht-granulomatöse, bakterielle Infektionen der Wirbelsäule ohne vorhergehende Operation und ohne einliegendes Implantat werden als pyogene Spondylodiszitis bezeichnet [27]. Die pyogene Spondylodiszitis ist eine potenziell lebensbedrohliche Infektion der Bandscheiben und angrenzenden Wirbelkörper mit einer in Europa steigenden Inzidenz [8]. Indolente Verläufe, insbesondere bei Infektionen mit niedrig virulenten Erregern führen häufig zu einer verzögerten Diagnosestellung, welche mit einer hohen Morbidität und Mortalität verbunden sein kann [20]. Nach wie vor gelten Staphylokokken, insbesondere *Staphyloccoccus aureus* (*S. aureus*) als die häufigsten infektverursachenden Erreger, gefolgt von gramnegativen Bakterien [11, 24]. Allerdings muss den Koagulase-negativen Staphylokokken („coagulase-negative staphylococci“ [CoNS]) eine zunehmende Bedeutung beigemessen werden [19, 26]. Diese sind bei (intravaskulären) katheter- und gerätebedingten Blutstrominfektionen relevant, welche eine hämatogene Spondylodiszitis auslösen können (fremdkörperassoziierte Infektionen) [26]. 

Vor dem Hintergrund der alternden Bevölkerung und der damit verbundenen Zunahme an medizinischen Interventionen wurde jüngst der Ätiologiebegriff der „healthcare“-assoziierten Spondylodiszitis etabliert [17, 22, 23]. Für die „healthcare“-assoziierte Spondylodiszitis wurden im Vergleich zur ambulant erworbenen Spondylodiszitis höhere Mortalitätsraten und Reinfektionsraten berichtet [23]. Bei der Auswahl einer empirischen antibiotischen Therapie ist die Kenntnis der Ätiologie, lokaler Erregerspektren und deren Resistenzprofile entscheidend. Die kalkulierte antibiotische Therapie ist insbesondere bei Patienten mit schweren oder progredienten neurologischen Ausfällen oder hämodynamischer Instabilität und im Fall von kulturnegativen Spondylodiszitiden erforderlich [4, 18]. In der aktuellen Leitlinie zur Diagnostik und Therapie der Spondylodiszitis wird die Wichtigkeit betont, die häufigsten Erreger mit der empirischen Therapie zu erfassen, eine spezifische Empfehlung zur Antibiotikaauswahl wurde allerdings nicht angegeben [13]. Ein sinnvoller und zielgerichteter Einsatz von Antibiotika ist notwendig, um Erreger mit einem möglichst schmalen Nebenwirkungsprofil zu adressieren. Außerdem muss die Ausbreitung von Antibiotikaresistenzen vermieden werden.

Ziel der vorliegenden Studie war es daher, 1) die bevorzugten empirischen Antibiotikaregime verschiedener orthopädisch-unfallchirurgischer Zentren an deutschen Universitäts- und Berufsgenossenschaftlichen Kliniken in der Behandlung der pyogenen Spondylodiszitis zu erheben und 2) die hypothetischen Sensibilitäts- und Resistenzraten für die angewandten empirischen Antibiotikatherapieschemata zu evaluieren.

## Studiendesign und Untersuchungsmethoden

Wir führten an deutschen Universitäts- und berufsgenossenschaftlichen Kliniken (BG-Kliniken), jeweils in den Fachbereichen Orthopädie und Unfallchirurgie, eine Umfrage zur empirischen Antibiotikatherapie durch: Im Januar 2021 wurden insgesamt 71 Kliniken per E‑Mail kontaktiert und um die Teilnahme an dieser Fragebogenaktion gebeten. Teilnahme-Erinnerungen wurden zweimal, im Abstand von 14 Tagen verschickt, um eine möglichst hohe Rekrutierungsrate zu erreichen. Der versendete Fragebogen beinhaltete offene Fragen zur empirischen antibiotischen Therapie bei pyogener Spondylodiszitis. Um eine hypothetische antibiotische Sensibilitäts- und Resistenzrate zu bestimmen, wurden die Umfrageergebnisse anschließend mit zuvor veröffentlichten, retrospektiven Daten zur antimikrobiellen Behandlung von *n* = 45 Spondylodiszitispatienten, die zwischen 2013 und 2020 in unserer unfallchirurgischen Klinik mit dezidiertem Fokus der Wirbelsäulenchirurgie behandelt wurden, verglichen [17]. Für diese Kohorten wurde die mikrobiologische Datenbank nach Erregern durchsucht, die aus Biopsien infizierter Wirbelsäulensegmente oder in Blutkulturen von Patienten mit Spondylodiszitis kulturell nachgewiesen worden waren. Die Biopsien wurden entweder CT-gesteuert oder offen chirurgisch gewonnen. Angezüchtete Bakterienisolate wurden mittels matrixunterstützter Laser-Desorption/Ionisation (MALDI-TOF-MS) auf Speziesebene identifiziert und einer Empfindlichkeitstestung im semiautomatisierten System (Becton Dickinson Phoenix System, Becton, Dickinson and Company, Franklin Lakes, NJ, USA) bzw. mit Agardiffusions-basierten Methoden unterzogen. Es lagen Antibiogramme bis in das Jahr 2013 zurückreichend vor. Die antimikrobiellen Empfindlichkeitstests wurden ab dem 2. Quartal 2018 nach den Richtlinien des European Committee on Antimicrobial Susceptibility Testing (EUCAST) durchgeführt [12], davor nach denen des Clinical Laboratory Standards Institute (CLSI) [7].

Die deskriptive und statistische Datenanalyse wurde mit der Software IBM SPSS Statistics durchgeführt (Version 28.0, IBM Corp, Armonk, NY, USA).

## Ergebnisse

### Bevorzugtes empirisches Antibiotikaregime in der Behandlung der pyogenen Spondylodiszitis

**Figure 1:**
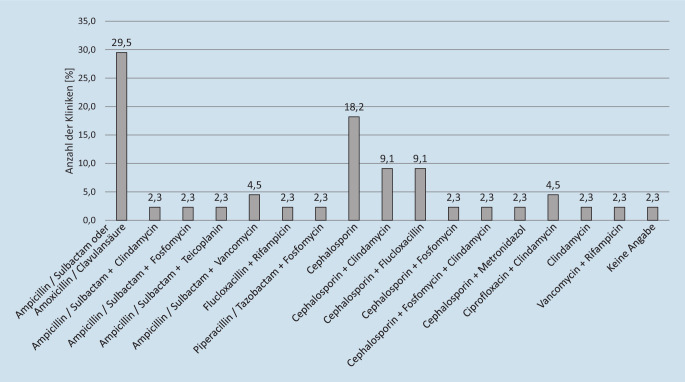

Von den 71 angefragten Kliniken antworteten insgesamt 44 (62,0 %). Die empirische antibiotische Behandlung bei Spondylodiszitis zeigte ein inhomogenes Therapiebild. Insgesamt wurden 16 verschiedene empirische Antibiotikatherapien als jeweiliger Standard berichtet. Eine Antwort enthielt keine spezifischen Angaben zu einer standardisierten antibiotischen Therapie. Am häufigsten wird eine Monotherapie mit einem Aminopenicillin + Betalaktamaseinhibitor verwendet (29,5 %). Am zweithäufigsten kommen Cephalosporine als Monotherapie zum Einsatz (18,2 %). Darüber hinaus wurden vor allem Kombinationstherapien als Therapiestandard angegeben (13 verschiedene Kombinationen), wobei am häufigsten ein Cephalosporin mit Clindamycin oder mit Flucloxacillin kombiniert wird (jeweils 9,1 %) (Tab. 1A; Abb. 1).

**Table 1:** 

A	Antworthäufigkeit				B
Antibiotika		Sensibilitätsrate [%]			Antibiotika
Amoxicillin/Clavulansäure o. Ampicillin/Sulbactam	13 (29,5 %)	77,8	95,6	1 (2,3 %)	Ampicillin/Sulbactam + Teicoplanin
Cephalosporin (Cefazolin/Ceftriaxon)	8 (18,2 %)	60,0	91,1	2 (4,5 %)	Ampicillin/Sulbactam + Vancomycin
Cephalosporin + Clindamycin	4 (9,1 %)	66,7	84,4	1 (2,3 %)	Ampicillin/Sulbactam + Clindamycin
Cephalosporin + Flucloxacillin	4 (9,1 %)	60,0	84,4	1 (2,3 %)	Piperacillin/Tazobactam + Fosfomycin
Ampicillin/Sulbactam + Vancomycin	2 (4,5 %)	91,1	82,2	1 (2,3 %)	Ampicillin/Sulbactam + Fosfomycin
Ciprofloxacin + Clindamycin	2 (4,5 %)	71,1	80,0	1 (2,3 %)	Vancomycin + Rifampicin
Ampicillin/Sulbactam + Teicoplanin	1 (2,3 %)	95,6	77,8	13 (29,5 %)	Amoxicillin/Clavulansäure o. Ampicillin/Sulbactam
Ampicillin/Sulbactam + Clindamycin	1 (2,3 %)	84,4	71,1	2 (4,5 %)	Ciprofloxacin + Clindamycin
Ampicillin/Sulbactam + Fosfomycin	1 (2,3 %)	82,2	66,7	4 (9,1 %)	Cephalosporin + Clindamycin
Flucloxacillin + Rifampicin	1 (2,3 %)	60,0	66,7	1 (2,3 %)	Cephalosporin + Fosfomycin + Clindamycin
Piperacillin/Tazobactam + Fosfomycin	1 (2,3 %)	84,4	64,4	1 (2,3 %)	Cephalosporin + Fosfomycin
Cephalosporin + Fosfomycin	1 (2,3 %)	64,4	64,4	1 (2,3 %)	Clindamycin
Cephalosporin + Fosfomycin + Clindamycin	1 (2,3 %)	66,7	60,0	8 (18,2 %)	Cephalosporin (Cefazolin/Ceftriaxon)
Cephalosporin + Metronidazol	1 (2,3 %)	60,0	60,0	4 (9,1 %)	Cephalosporin + Flucloxacillin
Clindamycin	1 (2,3 %)	64,4	60,0	1 (2,3 %)	Flucloxacillin + Rifampicin
Vancomycin + Rifampicin	1 (2,3 %)	80,0	60,0	1 (2,3 %)	Cephalosporin + Metronidazol

Sortierung der angegebenen Antibiotika nach Antworthäufigkeit (A) und nach potenzieller Sensibilitätsrate (B)

### Sensibilitäts- und Resistenzraten für die angewandten empirischen Antibiotikatherapieschemata

Die Umfrageergebnisse wurden mit Antibiogrammen einer zuvor analysierten Spondylodiszitiskohorte verglichen (*n* = 45, 56,8 % männlich, Alter 66,1 ± 12,4 Jahre) [17]. In dieser Kohorte wurden in 48,9 % der Fälle *S. aureus*, in 26,1 % CoNS, in 10,2 % Enterobacterales und in 6,8 % Streptokokken als auslösende Erreger nachgewiesen [17].

**Figure 2:**
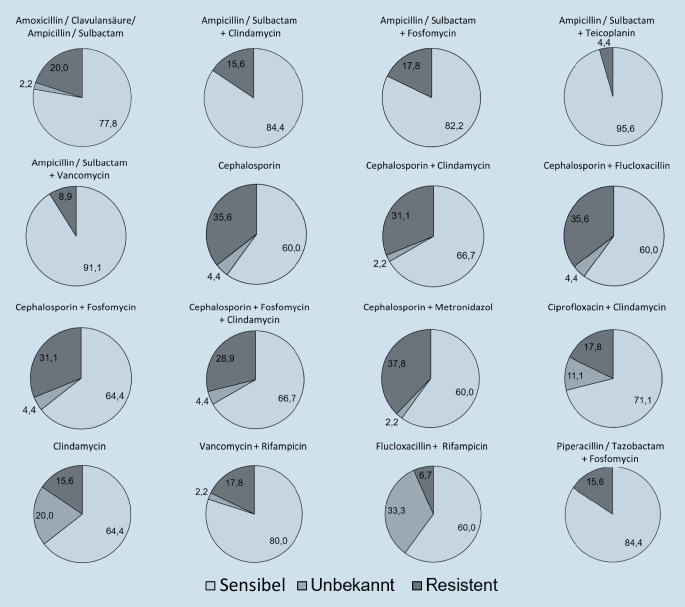

Anhand des Resistenzprofils der Erreger in 45 Fällen mit pyogener Spondylodiszitis konnte eine potenzielle Sensibilität für die als Standard angegebenen empirischen Substanzen abgeleitet werden. Die am häufigsten angegebene antibiotische Therapie, die Monotherapie mit Ampicillin/Sulbactam bzw. Amoxicillin/Clavulansäure würde demnach eine Sensibilitätsrate von 77,8 % erreichen. Durch eine Kombination mit Vancomycin oder Teicoplanin könnte die Sensibilitätsrate auf 91,1 % beziehungsweise auf 95,6 % gesteigert werden (Tab. 1B; Abb. 2). Die höchste potenzielle Resistenzrate zeigte sich für eine Monotherapie mit Cephalosporinen (35,6 % resistente Erreger). Die potenzielle Resistenzrate für Cephalosporine konnte durch eine Kombination mit Clindamycin (31,1 % resistente Erreger), Flucloxacillin (35,6 % resistente Erreger), Fosfomycin (31,1 % resistente Erreger), oder Metronidazol (37,8 % resistente Erreger) nicht relevant verbessert werden (Abb. 2). Eine dieser beiden Kombinationen wurde von drei Kliniken (6,8 %) als Standard angegeben. Hohe potenzielle Sensibilitätsraten würden außerdem mit einer Kombination aus Vancomycin + Piperacillin/Tazobactam (91,1 % sensible Erreger), Vancomycin + Meropenem (97,8 % sensible Erreger), Teicoplanin + Piperacillin/Tazobactam (100,0 % sensible Erreger) und Linezolid + Piperacillin/Tazobactam (100,0 % sensible Erreger) erreicht werden [17]. Diese Kombinationen wurden von keiner Klinik als Therapiestandard angegeben.

## Diskussion

Die aktuelle Umfrage zeigte eine große Heterogenität der standardmäßig angewandten kalkulierten antibiotischen Therapie bei pyogener Spondylodiszitis. Ein Abgleich mit 45 Antibiogrammen von Patienten, die bei pyogener Spondylodiszitis in unserer Klinik behandelt wurden, ergab eine hohe Diskrepanz zwischen den in der Umfrage häufig verwendeten empirisch eingesetzten Substanzen und potenziellen Antibiotikakombinationen mit einem breiten Wirkspektrum.

### Bevorzugte empirische Antibiotikaregime in der Behandlung der pyogenen Spondylodiszitis

Fehlende offizielle Richtlinien, lokale Unterschiede in den Resistenzprofilen und das heterogene klinische Erscheinungsbild der pyogenen Spondylodiszitis können Gründe für die mangelnde Einheit der in dieser Umfrage angegebenen empirischen antibiotischen Therapien sein.

Die am häufigsten genannten, empirischen Antibiotikatherapien mit Amoxicillin/Clavulansäure oder Ampicillin/Sulbactam (29,5 %) und mit Cephalosporinen (18,2 %) zeigten hohe potenzielle Resistenzraten von 20,0 % bzw. 35,6 % [17]. Hohe potenzielle antimikrobielle Abdeckungen konnten mit Vancomycin + Ampicillin/Sulbactam, mit Vancomycin + Piperacillin/Tazobactam, mit Ampicillin/Sulbactam + Teicoplanin und mit Vancomycin + Meropenem erreicht werden [17]. Diese Kombinationen decken die meisten grampositiven und gramnegativen Keime sowie Methicillin-resistente Erreger ab. Allerdings gaben insgesamt nur drei Kliniken (6,8 %) eine dieser Kombination als Standard an, welche eine potenzielle Sensibilitätsrate > 90,0 % erreichen würde. In einer Analyse von 101 Spondylodiszitisfällen von Desoutter et al. waren CoNS die häufigsten auslösenden Erreger (26 %) und stellten somit eine große Herausforderung für die empirische Therapie dar [10]. Die Resistenzrate aller Isolate gegenüber Amoxicillin/Clavulansäure beziehungsweise Ampicillin/Sulbactam betrug 17 % und gegenüber Cefazolin 32 %, ähnlich unseren Ergebnissen [10]. Hohe Resistenzraten wurden in der Studie von Desoutter et al. vor allem bei Enterobacteriaceae gefunden [10]. Eine Auswertung von 358 Spondylodiszitisfällen von Park et al. zeigte, vergleichbar mit unseren Ergebnissen hohe Resistenzraten für Monotherapien mit Amoxicillin/Clavulansäure (43,6 %) und Cefazolin (37,4 %) [22]. Auch in dieser Studie war eine gute Sensibilität (> 90 %) durch die Kombination mit Vancomycin zu erreichen, insbesondere bei „healthcare“-assoziierten Spondylodiszitiden [22].

Die Verwendung von Vancomycin birgt ein hohes Risiko für Nephrotoxizität, was zu einer hohen Morbidität und einem Therapieversagen führen kann [3]. Das ist insbesondere vor dem Hintergrund der wachsenden Anzahl an geriatrischen Patienten, mit oftmals bereits bestehenden chronischen Nierenerkrankungen kritisch zu sehen. Eine Überwachung des Vancomycin-Talspiegels bedeutet außerdem einen zusätzlichen Aufwand [29]. Verbunden mit der niedrigen Rate von Methicillin-resistenten *S.-aureus*-Infektionen könnten diese Aspekte Gründe dafür darstellen, dass Vancomycin häufig nicht routinemäßig als empirische Antibiose eingesetzt wird [30]. Bei der Entscheidung zum empirischen Einsatz von Vancomycin, zur Adressierung von CoNS gilt es zu beachten, dass diese fast immer nur bei Fremdkörperinfektionen eine Rolle spielen, welche in der Definition der „healthcare“-assoziierten Spondylodiszitis miterfasst sind [23, 26]. Hinweise auf eine solche Ätiologie sollten bei der Patientenanamnese erfragt werden und der empirische Einsatz von Vancomycin sollte fallspezifisch diskutiert werden.

### Sensibilitäts- und Resistenzraten für die angewandten empirischen Antibiotikatherapieschemata

Die Methicillin-Resistenz von *S. aureus* nahm in den letzten Jahren in Deutschland sukzessive ab [2, 30]. Im Gegensatz dazu muss den CoNS bei der Ätiologie der Spondylodiszitis eine wachsende Bedeutung zugemessen werden [6, 17, 19]. Das vorliegende Resistenzprofil aus der kürzlich durchgeführten Analyse kann zum einen durch das Resistenzmuster von CoNS erklärt werden, welche hohe Resistenzraten gegen β‑Lactam-Antibiotika, einschließlich Penicillin, Oxacillin/Methicillin, aber auch gegen Gentamicin, Clindamycin, Ciprofloxacin und Erythromycin aufweisen [18]. Da Methicillin-Resistenzen häufig sind, erfordern die meisten CoNS-Fälle den Einsatz von Zweitlinienantibiotika wie Vancomycin, Daptomycin oder Linezolid [21]: *Staphylococcus epidermidis*, weist oft eine Resistenz gegen Erstlinienantibiotika auf [9]. Gegenüber Vancomycin sind CoNS in der Regel sensibel [9]. Vancomycin ist daher als Bestandteil einer kombinierten empirischen Antibiotikatherapie bei Spondylodiszitis zu erwägen, insbesondere wenn Hinweise auf eine „healthcare“-assoziierte Spondylodiszitis vorliegen [17, 22].

Ein weiterer Erklärungsansatz für die niedrige antibiotische Abdeckung der auslösenden Erreger durch die in der Umfrage häufig genannten Cephalosporine ist das Resistenzprofil von *Escherichia coli* (*E. coli*). *E. coli* zeigte zuletzt in Deutschland eine signifikante Zunahme an Resistenzen gegenüber Cephalosporine der 3. Generation [2]. Wir wiesen zuletzt eine hohe Resistenzrate von gramnegativen Erregern gegen Vancomycin, Cefazolin und Ciprofloxacin nach [17]. In den jüngst veröffentlichten Antibiogrammen wies *E. coli* eine hohe Sensibilität gegenüber Piperacillin/Tazobactam und Meropenem auf [17]. Auch die Kombination dieser Präparate mit Vancomycin würde folglich zu einer sehr breiten antibiotischen Abdeckung führen. Die Verwendung von Carbapenemen sollte jedoch auf kritisch kranke Patienten beschränkt und nicht als empirische Therapie eingesetzt werden, da ihre Verwendung mit dem Risiko der Besiedlung mit antibiotikaresistenten Bakterien verbunden ist [28]. Vor dem Hintergrund der hohen Prävalenz von Multimorbidität der älter werdenden Bevölkerung in Deutschland [25] ist zu beachten, dass gramnegative Erreger häufiger in diesem Patientenkollektiv als auslösender Keim einer Spondylodiszitis identifiziert werden und mit einem schweren Krankheitsverlauf assoziiert sein können [14, 15].

Um das Risiko der Entwicklung multiresistenter Erreger zu minimieren, sollte unmittelbar mit erfolgtem Keimnachweis eine antibiogrammorientierte Umstellung auf Substanzen mit möglichst schmalem Spektrum und hoher oraler Bioverfügbarkeit und Knochenpenetration erfolgen [13, 18].

### Limitationen

Unsere Studie hat verschiedene Einschränkungen: Die Umfrage konzentrierte sich auf orthopädische und unfallchirurgische Abteilungen deutscher Universitätskliniken und BG-Kliniken. Die Auswahl der Krankenhäuser, die hoch spezialisierte Medizin auf diesem Gebiet anbieten, ist nicht zwangsläufig repräsentativ für den gesamten deutschen Krankenhaussektor. Dies betrifft naturgemäß auch das zu erwartende Erregerspektrum, gegen das diese Substanzen verglichen wurden. Dennoch wurde diese Auswahl getroffen, da davon ausgegangen wurde, dass das wissenschaftliche Interesse der eingeladenen Abteilungen eine hohe Rücklaufquote ermöglicht. Des Weiteren wurde davon ausgegangen, dass Universitätskliniken mit bestehenden kooperierenden Abteilungen für Infektionskrankheiten und Mikrobiologie, sich bei der Therapie an aktuellen Standards orientieren. Nach der Dauer der Antibiotikatherapie wurde nicht gefragt. Hierzu existieren allerdings, im Gegensatz zur Auswahl der für die empirische Therapie eingesetzten Substanzen, klare Empfehlungen [5, 18]. Außerdem wurde nicht nach der empirischen antibiotischen Therapie bei implantatassoziierten Infektionen der Wirbelsäule nach zuvor durchgeführten Operationen gefragt. Nicht zuletzt aufgrund der Notwendigkeit einer biofilmaktiven Therapie unterscheidet sich hierbei das therapeutische Vorgehen deutlich, sodass dazu nach Meinung der Autoren separate Untersuchungen und Umfragen durchgeführt werden sollten [16]. Der Abgleich der berichteten empirischen antibiotischen Therapien erfolgte mit einem zuvor veröffentlichten hypothetischen Resistenzprofil von Spondylodiszitiserregern aus 45 Fällen, welche über einen Zeitraum von 7 Jahren monozentrisch behandelt worden sind [17]. Dieses Profil ist aufgrund der zeitlichen Streuung und der geringen Fallzahl nicht abschließend repräsentativ für die aktuelle, deutschlandweite Resistenzsituation. Für diese Analyse und insbesondere für eine Formulierung einer Therapieempfehlung sind weitere, prospektive, multizentrische Auswertungen notwendig.

## Fazit für die Praxis

- Die deutschlandweite Umfrage zur kalkulierten antibiotischen Therapie bei pyogener Spondylodiszitis hat eine große Heterogenität ergeben.
- Die am häufigsten angegebenen Therapiestandards wiesen eine eingeschränkte Wirksamkeit in der Analyse anhand eines hypothetischen, monozentrischen Erregerresistenzprofils auf.
- Vorbehaltlich lokaler Erregerspektren und Resistenzprofile, sowie der individuellen Patientensituation, kann eine Kombination aus einem Breitspektrum-β-Laktam-Antibiotikum mit einem zusätzlichen Glykopeptidantibiotikum sinnvoll sein.
- Weitere, prospektive Studien zur Wirksamkeit und Sicherheit der empirischen Antibiotikakombinationstherapie könnten dazu beitragen, eine allgemeine Therapieempfehlung zu formulieren und die Qualität der empirischen Antibiotikatherapie bei pyogener Spondylodiszitis zu verbessern.

## Abkürzungen

BG: Berufsgenossenschaftlich
CLSI: Clinical Laboratory Standards Institute
CoNS: „Coagulase-negative staphylococci“
EUCAST: European Committee on Antimicrobial Susceptibility Testing
MALDI‐TOF‑MS: Matrixunterstützte Laser-Desorption/Ionisation-Massenspektrometrie
